# Overweight and Obesity and Associated Factors among School-Aged Adolescents in Six Pacific Island Countries in Oceania

**DOI:** 10.3390/ijerph121114505

**Published:** 2015-11-13

**Authors:** Supa Pengpid, Karl Peltzer

**Affiliations:** 1 ASEAN Institute for Health Development, Mahidol University, Salaya, Phutthamothon, Nkohn Pathom 73170, Thailand; E-Mail: supaprom@yahoo.com; 2 Department of Research & Innovation, University of Limpopo, Turfloop Campus, Sovenga 0727, South Africa; 3 HIV/AIDS/STIs and TB (HAST), Human Sciences Research Council, Pretoria 0001, South Africa

**Keywords:** overweight, obesity, overweight or obesity, dietary behaviour, physical activity, sedentary behaviour, psychosocial factors, soico-familial factors, global school-based health survey, Oceania

## Abstract

The aim of this study was to assess overweight and obesity and associated factors in school-going adolescents in six Pacific Island countries in Oceania. The sample included 10,424 school-going adolescents predominantly 13–16 years old from Fiji, Kiribati, Samoa, Solomon Islands, Tonga, and Vanuatu. Bivariate and multivariable analyses were conducted to assess the relationship between dietary behaviour, substance use, physical activity, psychosocial factors, social-familial influences, and overweight or obesity. The prevalence of overweight and obesity was determined based on self-reported height and weight and the international child body mass index standards. Results indicate a prevalence of overweight or obesity of 24.3% and obesity of 6.1% in the six countries, ranging in terms of overweight or obesity and obesity from 12.0% and 0.4% in Vanuatu to 58.7% and 21.1% in Tonga, respectively. In multivariable regression analysis, being female was associated with overweight, carbonated soft drink use with obesity, sedentary behaviour with overweight or obesity, suicidal ideation with overweight, having close friends and peer support with overweight and obesity, parental or guardian supervision with overweight, and parental or guardian bonding was associated with overweight or obesity. High prevalence rates of overweight and obesity were found and several factors identified which can help guide interventions.

## 1. Introduction

The prevalence of adolescent overweight and obesity seems to have been substantially increasing worldwide [1], including in low-income and middle-income countries [1], with a prevalence of 10%–20% in south-east Asian and western Pacific regions [2]. While there are studies reporting high rates of adult obesity in the Pacific Island countries in Oceania [3,4,5,6], fewer studies are found on childhood obesity. In secondary school children (age 12–18 years) in Fiji 16.7% were overweight and 5.2% obese [7]. The prevalence of obesity in Tonga children (12–19 years) was 6.0% to 22.8% among girls and 2.2%–3.8% among boys depending on Island of residence and obesity classification [8], and in Vanuata 3%–6% in three islands overweight or obesity among boys and 0–11.4% among girls [9]. Ng *et al.* [10] estimated as part of the Global Burden of Disease Study 2013, a prevalence of overweight and obesity in Fiji 12.8% among boys (<20 years) and 24.9% among girls; 47.7% and 66.1% among boys and girls, respectively, in Kiribati; 42.2% and 50.0% among boys and girls, respectively, in Samoa; 28.3% and 49.2% among boys and girls, respectively, in Solomon Islands; 34.5% and 52.6% among boys and girls, respectively, in Tonga; and 14.5% and 23.2% among boys and girls (<20 years), respectively, in Vanuatu.

Childhood overweight and obesity have detrimental health consequences during childhood and adulthood [11]. In order to plan services for the provision of care and to evaluate the impact of policy strategies it is important to monitor the prevalence of obesity [12]. 

Factors identified in previous studies to be associated with overweight and/or obesity in adolescents include: (1) sociodemographic factors: male gender [13,14], female gender [15,16], younger age [13]; (2) dietary factors: sugar-sweetened beverages [17], frequently consumed fast food [18], lack of fruit consumption [19], not feeling hungry [16]; (3) Physical inactivity and high sedentary behavior [20]; (4) Other health risk behavior such as bullying victimization [21], smoking [22,23]; (5), psychosocial factors [24,25], including familial influences such as authoritative parenting style [26]. Some of these risk factors for adolescent obesity identified may differ in the Oceania study region. Therefore, the aim of this study was to assess overweight and obesity and associated factors in school-going adolescents in six Pacific Island countries in Oceania.

## 2. Methods

### 2.1. Description of Survey and Study Population

This study involved secondary analysis of existing data from the Global School-Based Health Survey (GSHS) from six Pacific Island countries in Oceania (Fiji 2010, Kiribati 2011, Samoa 2011, Solomon Islands 2011, Tonga 2010, and Vanuatu 2011). All Pacific Island countries from which GSHS datasets with the two-stage cluster sample design and Body Mass Index (BMI) information were publicly available were included in this secondary analysis. Details and data of the GSHS can be accessed [27] A two-stage cluster sample design was used to collect data to represent all students in grades 6, 7, 8, 9, and 10 in the country. At the first stage of sampling, schools were selected with probability proportional to their reported enrollment size. In the second stage, classes in the selected schools were randomly selected and all students in selected classes were eligible to participate irrespective of their actual ages. Students self-completed the questionnaires to record their responses to each question on a computer scanable answer sheet. The GSHS 10 core questionnaire modules address the leading causes of morbidity and mortality among children and adults worldwide: tobacco, alcohol and other drug use; dietary behaviors; hygiene; mental health; physical activity; sexual behaviors that contribute to HIV infection, other sexually transmitted infections, and unintended pregnancy; unintentional injuries and violence; protective factors and respondent demographics [27].

### 2.2. Measures

#### 2.2.1. BMI Measurement and Overweight Classification

Body weight and height were assessed by self-report. School children with BMI figures relating to an adult BMI of <25.0 kg/m^2^ were categorized as having normal weight and those with a BMI of ≥25.0 kg/m^2^ were categorized as having overweight, while those with a BMI value of ≥30.0 kg/m^2^) were classified as obese. For the BMI cut-points used to define the different weight classifications, international age- and gender-specific criteria were used [28].

#### 2.2.2. Dietary Behaviour and Substance Use

Carbonated soft drinks: “During the past 30 days, how many times per day did you usually drink carbonated soft drinks such as Pepsi, Seven-up, Fanta, Coke, *etc*.” Response options ranged from 0 = I did not drink any carbonated soft drinks in the past 30 days to 6 = 5 or more times per day [27]. 

Eating food from a fast food restaurant: “During the past seven days, on how many days did you eat food from a fast food restaurant, such as such country specific examples?” Response options ranged from 0 to 7 days [27].

Fruits: “During the past 30 days, how many times per day did you usually eat fruit, such as ‘country specific examples’?” Response options ranged from 1 = I did not eat fruit during the past 30 days to 7 = 5 or more times per day [27].

Vegetables: “During the past 30 days, how many times per day did you usually eat vegetables, such as ‘country specific examples’?” Response options ranged from 1 = I did not eat vegetables during the past 30 days to 7 = 5 or more times per day [27]. 

Hunger: “During the past 30 days, how often did you go hungry because there was not enough food in your home?” Response options ranged from 1 = never to 5 = always [27].

Substance Use. Smoking cigarettes: “During the past 30 days, on how many days did you smoke cigarettes?” Response options ranged from 1 = 0 day to 7 = all 30 days [27].

Alcohol use: “During the past 30 days, on how many days did you have at least one drink containing alcohol?” Response options ranged from 1 = 0 day to 7=all 30 days [27].

#### 2.2.3. Physical Activity and Sedentary ÿEhavior

Leisure time physical activity was assessed with two questions: “During the past 7 days, on how many days were you physically active for a total of at least 60 min. per day?” and “During a typical or usual week, on how many days are you physically active for a total of at least 60 min per day?” Physical inactivity was classified as four or less days at least 60 min. per day physically active in a week [27]. Students were also asked about how many days they had walked or rode a bicycle to or from school during the past seven days, and how many times they had attended a physical education class during each week during the school year [27]. 

Leisure time sedentary behavior: “How much time do you spend during a typical or usual day sitting and watching television, playing computer games, talking with friends, or doing other sitting activities?” [27].

#### 2.2.4. Psychosocial Factors and Socio-Familial Influences

(1) *Loneliness*: “During the past 12 months, how often have you felt lonely?” (Response options ranged from 1 = never to 5 = always). (2) *Anxiety or worried:* “During the past 12 months, how often have you been so worried about something that you could not sleep at night?” (Response options ranged from 1 = never to 5 = always). (3) *Suicidal ideation*. “During the past 12 months, did you make a plan about how you would attempt suicide?” (Response option was yes or no). (4) *No close friends*: “How many close friends do you have?” (Response options ranged from 1 = 0 to 4 = 3 or more). (5) *Bullied:* Having been bullied at least once in the preceding 30 days, was classified as bullied [27]. Socio-familial influences were assessed with questions on peer support at school, parental or guardian supervision, connectedness, and bonding. Peer support at school was assessed with the question: “During the past 30 days, how often were most of the students in your school kind and helpful?” Parental or guardian supervision: “During the past 30 days, how often did your parents or guardians check to see if your homework was done?” Parental or guardian connectedness: “During the past 30 days, how often did your parents or guardians understand your problems or worries?” and Parental or guardian bonding: “During the past 30 days, how often did your parents or guardians really know what you were doing with your free time?” (Response options to these questions were from 1 = never to 5 = always) [27]. 

#### 2.2.5. Data Analysis

Data analysis was performed using STATA software version 13.0 (Stata Corporation, College Station, TX, USA). This software has the advantage of directly including robust standard errors that account for the sampling design, *i.e.* cluster sampling owing to the sampling of school classes. Associations between dietary behaviour and substance use, physical activity, psychosocial, and socio-familial factors and overweight, obesity and overweight or obesity among school children were evaluated calculating odds ratios (OR). Unconditional logistic regression was used for evaluation of the impact of explanatory variables for overweight or obesity and obesity (binary dependent variables). All variables statistically significant at the *p* < 0.05 levels in bivariate analyses were included in the multivariable models. In the analysis, weighted percentages are reported. The reported sample size refers to the sample that was asked the target question. The two-sided 95% confidence intervals are reported. The *p* values less or equal to 5% are used to indicate statistical significance. Both the reported 95% confidence intervals and the *p* value are adjusted for the multi-stage stratified cluster sample design of the study.

## 3. Results

### 3.1. Sample Characteristics

The study response rate for the Fiji was 90%, Kiribati 85%, Samoa 79%, Solomon Islands 85%, Tonga 80%, and Vanuatu 72%. The final sample included 10,424 school-going adolescents predominantly 13–16 years old from Fiji, Kiribati, Samoa, Solomon Islands, Tonga, and Vanuatu; Kiribati, Samoa, Solomon Islands and Vanuatu are lower middle income countries and Fiji and Tonga upper lower middle income countries [29]. The study found a prevalence of overweight or obesity of 24.3% and obesity of 6.1% in six pacific island countries in Oceania. There was a large country variation in terms of overweight or obesity and obesity ranging from 12.0% and 0.4% in Vanuatu to 58.7% and 21.1% in Tonga, respectively. In terms of dietary behaviour and substance use, 40.3% consumed one or more carbonated soft drinks per day, 24% had three or more times fast foods per week, 49% had two or more fruits per day, 34.9% three of more servings of vegetables per day, 18.7% has consumed alcoholic beverages in the past month and 23.3% were current tobacco users. Regarding physical activity, 67.6% were physically inactive (less than 60 min/day), 39.2% did not walk or bike to school, 22.3% spent three or more hours sitting in a day, and 32.4% engaged in three or more days of physical education in a week. Regarding psychosocial factors, being bullied was the most frequent one, followed by suicidal ideation and having no close friends. Socio-familial influences were highest for parental or guardian supervision and peer support (see Table 1).

**Table 1:** Descriptive data of school-going adolescents aged predominantly 13–16 years (N = 10,424).

Variable	Sample	Overweight	Obesity	Overweight or Obesity
	N (%)	% (95% CI)	% (95% CI)	% (95% CI)
All	10424	18.2 (16.2–20.5)	6.1 (5.1–7.2)	24.3 (21.7–27.2)
Fiji	1773 (16.0)	13.9 (11.4–16.8)	5.2 (4.0–6.7)	19.1 (16.0–22.5)
Kiribati	1582 (15.2)	32.5 (29.6–35.4)	7.4 (5.7–9.6)	39.9 (36.5–43.4)
Samoa	2418 (23.2)	32.6 (30.0–35.4)	19.3 (16.8–22.2)	52.0 (48.3–55.6)
Solomon Islands	1421 (13.6)	18.9 (15.0–23.6)	2.7 (1.6–4.4)	21.6 (17.0–27.0)
Tonga	2211 (21.2)	37.6 (35.7–39.5)	21.1 (18.6–23.8)	58.7 (55.9–61.4)
Vanuatu	1119 (10.7)	11.5 (7.5–17.3)	0.4 (0.1–1.4)	12.0 (7.7–18.0)
*Country income*				
Kiribati, Samoa, Solomon Islands and Vanuatu	6540 (62.7)	20.5 (17.5–23.9)	5.0 (3.7–6.8)	25.6 (21.5–30.1)
Fiji and Tonga	3884 (37.3)	16.4 (13.9–19.4)	6.9 (5.9–8.4)	23.3 (20.0–27.0)
*Gender*				
Female	5672 (49.4)	21.0 (18.3–23.9)	6.0 (4.9–7.3)	27.0 (23.7–30.1)
Male	4566 (50.6)	15.5 (13.5–17.8)	6.2 (4.9–7.7)	21.7 (18.8–24.8)
*Age in years*				
13 or less	2725 (30.2)	15.8 (12.0–20.5)	5.8 (4.3–7.8)	21.5 (17.1–26.8)
*Age in years*	N (%)	% (95% CI)	% (95% CI)	% (95% CI)
14	3282 (25.9)	19.0 (16.4–22.1)	7.7 (6.4–9.2)	26.7 (23.1–30.7)
15	2934 (24.7)	19.5 (16.9–22.3)	5.9 (4.7–7.2)	25.4 (22.3–28.7)
16 or more	1364 (19.2)	19.1 (15.6–23.0)	3.9 (2.8–5.6)	23.0 (19.2–27.2)
*Dietary behaviour and substance use*				
One or more carbonated soft drinks per day	3974 (40.3)	23.9 (20.3–27.9)	8.1 (6.1–10.5)	31.2 (26.4–36.4)
Fast foods three or more times per week	2278 (24.0)	23.9 (20.3–27.9)	7.6 (5.7–10.0)	31.5 (26.8–36.6)
Fruits 2+ a day	4582 (49.0)	17.9 (15.3–20.9)	5.7 (4.5–7.3)	23.4 (20.7–27.0)
Vegetables 3+ a day	3444 (34.9)	18.2 (15.6–21.1)	5.9 (4.8–7.4)	24.1 (21.0–27.5)
Most of the time or always hunger	1761 (11.7)	20.4 (16.6–24.8)	6.9 (4.9–9.6)	27.2 (22.5–32.6)
Alcohol use in past month	2270 (18.7)	17.8 (14.7–21.4)	7.1 (5.3–9.4)	24.9 (20.9–29.5)
Tobacco use in past month	2549 (23.3)	19.4 (15.9–23.4)	8.6 (6.7–11.0)	28.0 (23.6–32.8)
*Physical activity*				
Physical activity less than 60 min per day on at least five days per week	7283 (67.6)	18.6 (16.2–21.3)	6.4 (5.3–7.8)	25.0 (22.0–28.3)
Not walk or bike to school at least once a week	4219 (39.2)	17.6 (15.2–20.3)	5.9 (4.7–7.4)	23.5 (20.1–27.2)
Sedentary behaviour (three hours or more a day)	2698 (22.3)	19.6 (17.0–22.5)	6.8 (5.4–8.5)	26.4 (23.0–30.0)
Physical education three or more days a week	2940 (32.4)	19.9 (17.6–22.3)	5.3 (3.9–7.1)	25.2 (22.1–28.5)
*Psychosocial factors*				
Loneliness	1505 (11.3)	17.9 (14.6–21.8)	7.0 (4.8–9.9)	24.9 (20.7–29.5)
Anxiety or worried	1614 (10.7)	18.9 (15.1–23.4)	6.7 (4.7–9.3)	25.5 (20.1–31.8)
Suicidal ideation	2072 (24.1)	21.0 (18.0–24.4)	5.8 (4.2–8.1)	26.8 (22.9–31.2)
No close friends	1018 (12.4)	14.2 (11.1–17.9)	8.0 (5.1–12.4)	22.2 (17.3–28.1)
Being bullied	5257 (61.6)	18.4 (16.2–20.9)	5.9 (4.4–7.9)	24.3 (20.9–28.0)
*Social–familial factors*				
Peer support (most of the time or always)	2733 (29.7)	24.5 (20.9–28.5)	8.6 (6.7–11.1)	33.1 (28.2–38.4)
Parental or guardian supervision (most of the time or always)	2964 (34.5)	25.2 (21.7–29.1)	8.1 (6.3–10.3)	33.3 (28.5–38.6)
Parental or guardian connectedness (most of the time or always)	2987 (23.6)	23.8 (19.5–28.6)	7.6 (5.6–10.3)	31.1 (25.3–38.0)
Parental or guardian bonding (most of the time or always)	2425 (27.4)	25.4 (21.8–29.3)	8.1 (6.4–10.2)	33.5 (28.8–38.5)

### 3.2. Association with Overweight, Obesity, and Overweight or Obesity

In multivariate analysis, being female was associated with overweight and overweight or obesity but not with obesity. Carbonated soft drink use was associated with obesity. Sedentary behaviour was associated with overweight or obesity, and having three or more days a week physical education was associated with overweight. Psychosocial factors (suicidal ideation) were associated with overweight and overweight or obesity but not with obesity. In terms of social-familial factors, having close friends and peer support was associated with overweight and obesity, respectively, parental or guardian supervision was associated with overweight, and parental or guardian bonding was associated with overweight or obesity. Further, in bivariate analyses, tobacco use was associated with obesity and living in an upper middle country was marginally significantly associated with obesity. Other dietary behaviour (eating vegetables, hunger), alcohol use, physical activity, and other psychosocial factors (anxiety, loneliness, being bullied) were not found to be associated with overweight, obesity and overweight or obesity (see Table 2 and Table 3).

**Table 2:** Overweight and obesity regression model.

Variable	Overweight		Obese	
	OR ^1^ (95% CI)	AOR ^2^ (95% CI)	OR ^1^ (95% CI)	AOR ^2^ (95% CI)
*Gender*				
Female	1.00	1.00	1.00	–––
Male	0.69 (0.59–0.81) ***	0.62 (0.50–0.77) **	1.03 (0.78–1.36)	
*Age in years*				
13 or less	1.00	–––	1.00	–––
14	1.26 (0.91–1.74)		1.35 (0.98–1.87)	
15	1.29 (0.91–1.84)		1.01 (0.71–1.45)	
16 or more	1.26 (0.85–1.86)		0.67 (0.41–1.09)	
*Country income*				
Lower Middle Income Country	1.00	–––	1.00	–––
Upper Middle Income Country	0.76 (0.58–1.01)		1.40 (0.95–2.04)	
*Dietary behaviour and substance use*				
One or more carbonated soft drinks per day	1.06 (0.92–1.23)	–––	1.32 (1.09–1.61) **	1.29 (1.08–1.54) **
Fast foods three or more times per week	1.10 (0.92–1.32)	–––	1.12 (0.89–1.41)	–––
Fruits 2+ a day	0.97 (0.77–1.22)	–––	0.91 (0.72–1.15)	–––
Vegetables 3+ a day	0.99 (0.86–1.15)	–––	0.97 (0.77–1.22)	–––
Most of the time or always hunger	1.17 (0.94–1.47)	–––	1.16 (0.83–1.62)	–––
Alcohol use in past month	1.02 (0.83–1.26)	–––	1.23 (0.88–1.71)	–––
Tobacco use in past month	1.02 (0.83–1.26)	–––	1.36 (1.00–1.83) *	1.02 (0.78–1.32)
*Physical activity*				
Physical activity less than 60 min per day on at least five days per week	1.09 (0.91–1.32)	–––	1.29 (0.94–1.76)	–––
Not walk or bike to school at least once a week	0.93 (0.77–1.12)	–––	0.95 (0.73–1.24)	–––
Sedentary behaviour (three hours or more per day)	1.14 (1.00–1.29) *	1.03 (0.82–1.29)	1.19 (0.93–1.52)	–––
Physical education three or more days/week	1.18 (1.03–1.35) *	1.23 (1.00–1.50) *	0.83 (0.61–1.11)	–––
*Psychosocial factors*				
Loneliness	1.00 (0.81–1.22)	–––	1.18 (0.83–1.68)	–––
Anxiety or worried	1.05 (0.82–1.34)	–––	1.13 (0.76–1.67)	–––
Suicidal ideation	1.42 (1.21–1.66) ***	1.44 (1.14–1.81) **	1.26 (0.89–1.78)	–––
No close friends	0.72 (0.56–0.92) **	0.50 (0.43–0.80) ***	1.40 (0.86–2.20)	–––
Being bullied	1.05 (0.88–1.25)	–––	0.94 (0.63–1.40)	–––
*Social–familial factors*				
Peer support (most of the time or always)	1.17 (0.97–1.41)	–––	1.45 (1.16–1.81) ***	1.43 (1.12–1.83) **
Parental or guardian supervision (most of the time or always)	1.26 (1.07–1.50) **	1.23 (1.00–1.50) *	1.13 (1.10–1.83) **	1.08 (0.86–1.35)
Parental or guardian connectedness (most of the time or always)	1.11 (0.89–1.39)	–––	1.16 (0.86–1.58)	–––

**^1^** OR = Odds Ratio; **^2^** AOR = Adjusted Odds Ratio; CI = Confidence Interval. *** *p* < 0.001; ** *p* < 0.01; * *p* < 0.05.

**Table 3:** Overweight or obesity regression model.

Variable	Overweight or Obese			
	OR ^1^ (95% CI)	AOR ^2^ (95% CI)
*Gender*		
Female	1.00	1.00
Male	0.75 (0.62–0.89) ***	0.63 (0.53–0.74) ***
*Age in years*		
13 or less	1.00	–––
14	1.33 (0.99–1.78)	
15	1.24 (0.90–1.69)	
16 or more	1.09 (0.76–1.56)	
*Country income*		
Lower Middle Income Country	1.00	–––
Upper Middle Income Country	0.89 (0.66–1.19)	
*Dietary behaviour and substance use*		
One or more carbonated soft drinks per day	1.15 (0.99–1.34)	–––
Fast foods three or more times per week	1.13 (0.96–1.32)	–––
Fruits 2+ a day	0.95 (0.80–1.12)	–––
Vegetables 3+ a day	0.98 (0.85–1.14)	–––
Most of the time or always hunger	1.19 (0.95–1.49)	–––
Alcohol use in past month	1.05 (0.88–1.26)	–––
Tobacco use in past month	1.13 (0.93–1.37)	–––
*Physical activity*		
Physical activity less than 60 min per day on at least five days per week	1.16 (0.99–1.36)	–––
Not walk or bike to school at least once a week	0.93 (0.77–1.13)	–––
Sedentary behaviour (three hours of more per day)	1.17 (1.03–1.34) *	1.21 (1.01–1.46) *
Physical education three or more days/week	1.08 (0.94–1.25)	–––
*Psychosocial factors*		
Loneliness	1.05 (0.88–1.26)	–––
Anxiety or worried	1.08 (0.81–1.45)	–––
Suicidal ideation	1.42 (1.24–1.63) ***	1.38 (1.13–1.68) **
No close friends	0.87 (0.66–1.16)	–––
Being bullied	1.02 (0.85–1.22)	–––
*Social–familial factors*		
Peer support (most of the time or always)	1.28 (1.08–1.53) **	1.06 (0.86–1.31)
Parental or guardian supervision (most of the time or always)	1.33 (1.13–1.58) ***	1.17 (0.94–1.45)
Parental or guardian connectedness (most of the time or always)	1.14 (0.89–1.47)	–––
Parental or guardian bonding (most of the time or always)	1.31 (1.12–1.54) ***	1.29 (1.06–1.56) **

^1^ OR = Odds Ratio; ^2^ AOR = Adjusted Odds Ratio; CI = Confidence Interval. *** *p* < 0.001; ** *p* < 0.01; * *p* < 0.05.

## 4. Discussion

The study found a high prevalence of overweight or obesity of 24.3% among school-going adolescents (27.0% in girls and 21.7% in boys) across six Pacific Island countries in Oceania, ranging from 12.0% in Vanuatu to 58.7% in Tonga. The overall prevalence of overweight or obesity and country variation, compare largely with previous studies in the region [7,8,9,10], is higher than in developing countries in general (13.4% in girls 12.9% in boys) and similar to overweight or obesity rates among children and adolescents in developed countries (22.6% in girls and 23.8% in boys) [10]. Some studies [30,31] found that overweight was positively related to national income, while in this study higher country income status was only marginally significantly associated with obesity and not overweight. Country variation in the prevalence of adolescent obesity may be related to differences in the degree of rapid social change [32] and sociocultural factors related to dietary patterns, physical activity, and body size [33] needing further investigation. 

This study found a higher prevalence rate of overweight and overweight or obesity in female than male adolescents, which concurs with studies among adolescents in low and middle income countries e.g., [10,15,16]. Possible reasons for this may be cultural differences in beliefs regarding body image. McCabe *et al.* [34] found that Fijian and Tongan adolescents were more likely to value a large body [32]. Further, Poskitt [35] found that in societies in transition, girls may be more susceptible to overweight than boys during the health transition, as they grow up. Interestingly, however, that overall there was no significant gender difference in the prevalence of obesity in this study. However, in lower middle income study countries (Kiribati, Samoa, Solomon Islands and Vanuatu) the prevalence of obesity was significantly higher in girls (5.7%) than in boys (4.4%), while in upper middle income study countries (Fiji and Tonga) the prevalence of obesity was significantly higher in boys (7.6%) than in girls (6.2%) (analysis not shown). This finding concurs with some studies e.g., [36] in most high income countries in Europe, with boys having higher rates of overweight or obesity than girls.

Further, regarding dietary behaviour, this study found an association between the consumption of sugar-sweetened beverages (SSBs) and obesity, which is confirmed from a majority of studies in a review [17]. SSBs are the primary source of added sugar in the diet of children and adolescents [17]. A high per capita soft drink consumption in Pacific Island countries and territories has also been confirmed by trade data [37]. Although this study did not find an association between physical inactivity and overweight and obesity, high sedentary behaviour was found to be associated with overweight or obesity, as also found in some previous studies [20]. In bivariate analysis tobacco use was associated with obesity, which is conforming to some previous studies [22,23]. There may be a belief that adolescents, particularly girls, use smoking as a means to control body weight [23].

In terms of psychosocial factors, having suicidal ideation was significantly associated with overweight and overweight or obesity but not with obesity in this study. Zeller *et al.* [38] found that relative to healthy weight, being obese was associated with significantly greater risk for adolescent engagement in suicidal ideation, but was unrelated to suicide attempts. The importance of linking psychosocial stressors and childhood obesity has been emphasized [39].

A positive parenting style (parental or guardian supervision and connectedness) were found in this study to be associated with overweight and overweight or obesity, respectively, while a previous study found a negative parenting style (authoritarian parents) was associated with childhood obesity [26]. Moreover, having close friends and positive peer support was found to be associated with overweight and obesity, respectively. It is possible that socio-cultural values of body size contribute to this. Similarly, Petersen *et al.* [7] found a positive association between the psychosocial aspect of health-related quality of life and obesity in secondary school children in Fiji. Traditionally, some groups in Fiji perceive a large body size as desirable and also of being cared for [7]. More research is needed to investigate social-familial influences on childhood obesity.

Unlike previous studies [18,19,20], this study did not find an association between physical inactivity, not walking or cycling to school, specific dietary behaviours such as frequently consumed fast food and lack of fruit consumption and overweight and obesity. It is possible that because of the high prevalence of physical inactivity, no association was found. 

## 5. Study Strength and Limitations

A strength of the use of GSHS was standardized methods and questionnaires were used across study countries. Further, a validation study of the health-risk behaviour component of the GSHS questionnaire found good validity in one of the Oceania study countries (Fiji) [40]. The study survey was cross-sectional and therefore no causal inferences can be made. The cut-offs used with self-reported BMI may lead to underestimation or overestimation of overweight and obesity [38]. The BMI was assessed by self-reported weight and height and could have included anthropometry to evaluate weight status. Dietary behaviour was not assessed with a validated food questionnaire, which may be included in future studies. Moreover, for the assessment of some concepts such as psychosocial factors, only one-item measures were used. In addition, several factors such as body composition (body fat) known to be associated with obesity status were not assessed.

## 6. Conclusions

High prevalence rates of overweight or obesity were found among school-going adolescents in six Pacific Island countries in Oceania. Increased strategies are needed to prevent and treat overweight and obesity in youth. 
